# The Combination of the CDK4/6 Inhibitor, Palbociclib, With the Vitamin D_3_ Analog, Inecalcitol, Has Potent *In Vitro* and *In Vivo* Anticancer Effects in Hormone-Sensitive Breast Cancer, But Has a More Limited Effect in Triple-Negative Breast Cancer

**DOI:** 10.3389/fendo.2022.886238

**Published:** 2022-06-17

**Authors:** Justine Vanhevel, Lieve Verlinden, Shauni Loopmans, Stefanie Doms, Iris Janssens, Sien Bevers, Steve Stegen, Hans Wildiers, Annemieke Verstuyf

**Affiliations:** ^1^ Laboratory of Clinical and Experimental Endocrinology, Department of Chronic Diseases and Metabolism (CHROMETA), Katholieke Universiteit (KU) Leuven, Leuven, Belgium; ^2^ Department of General Medical Oncology and Multidisciplinary Breast Center Leuven, University Hospitals (UV) Leuven, Leuven, Belgium

**Keywords:** vitamin D_3_ analog, palbociclib, breast cancer, cell proliferation and survival, mitochondria

## Abstract

Active vitamin D_3_, 1,25-dihydroxyvitamin D_3_ [1,25(OH)_2_D_3_], and its synthetically derived analogs possess potent anticancer properties. In breast cancer (BC) cells, 1,25(OH)_2_D_3_ blocks cell proliferation and induces apoptosis through different cell-type specific mechanisms. In this study, we evaluated if the combination of the potent vitamin D_3_ analog, inecalcitol, with a selective CDK4/6 inhibitor, palbociclib, enhanced the antiproliferative effects of both single compounds in hormone-sensitive (ER^+^) BC, for which palbociclib treatment is already approved, but also in triple-negative BC (TNBC). Inecalcitol and palbociclib combination treatment decreased cell proliferation in both ER^+^ (T47D-MCF7) and TNBC (BT20-HCC1143-Hs578T) cells, with a more pronounced antiproliferative effect in the former. In ER^+^ BC cells, the combination therapy downregulated cell cycle regulatory proteins (p)-Rb and (p)-CDK2 and blocked G1-S phase transition of the cell cycle. Combination treatment upregulated p-mTOR and p-4E-BP1 protein expression in MCF7 cells, whereas it suppressed expression of these proteins in BT20 cells. Cell survival was decreased after inecalcitol treatment either alone or combined in MCF7 cells. Interestingly, the combination therapy upregulated mitochondrial ROS and mitotracker staining in both cell lines. Furthermore, *in vivo* validation in a MCF7 cell line-derived xenograft mouse model decreased tumor growth and cell cycle progression after combination therapy, but not in a TNBC BT20 cell line-derived xenograft model. In conclusion, we show that addition of a potent vitamin D_3_ analog to selective CDK4/6 inhibitor treatment results in increased antiproliferative effects in ER^+^ BC both *in vitro* and *in vivo*.

## 1 Introduction

The vitamin D receptor (VDR), a member of the class II nuclear receptor superfamily, acts as a ligand-dependent transcription factor upon binding to the biologically active form of vitamin D, 1α,25-dihydroxyvitamin D_3_ [1,25(OH)_2_D_3_]. The VDR is present in classical target tissues such as kidney, bone and intestine, but also in many other (cancerous) tissues (1). By binding to the VDR, 1,25(OH)_2_D_3_ regulates calcium and phosphate homeostasis in the body, and, additionally has potent antiproliferative and pro-differentiating actions on various normal as well as cancerous cell types such as breast cancer (BC) cells (2). Previous work by our and other groups has demonstrated that treatment of BC cells with 1,25(OH)_2_D_3_ inhibits BC cell proliferation by hampering the transition from G0/G1 to S phase through induction of cyclin-dependent kinase inhibitors (CDKIs) such as *CDKN2D (p19)*, *CDKN1A (p21)*, and *CDKN1B (p27)* (3). However, to induce these antiproliferative effects, supraphysiological doses of 1,25(OH)_2_D_3_ are needed, which cause calcemic side effects *in vivo* such as hypercalcemia, hypercalciuria and hyperphosphatemia. To address this problem, more potent vitamin D_3_ analogs were developed such as inecalcitol (TX522), a vitamin D_3_ analog with increased antiproliferative capacity and reduced calcemic activity compared with natural 1,25(OH)_2_D_3_ (1).

With 2.3 million diagnoses each year, BC is the most frequently diagnosed cancer in women and the leading cause of cancer-related mortality worldwide (4, 5). BC is a heterogenous disease that can be subdivided into different subtypes based on the expression of hormonal and other receptors, such as the estrogen receptor (ER), the progesterone receptor (PR) and the human epidermal growth factor receptor 2 (HER2) (6). These markers allow classification of BC into hormonal receptor positive, HER2^+^ and triple-negative BC (TNBC), which do not express any of these receptors. Dysregulation of cell proliferation is one of the defined hallmarks of cancer development and several genetic alterations in key cell cycle regulatory genes have been described in BC. Hence, small-molecule inhibitors of cyclin D kinases (CDK) 4 and 6, such as palbociclib (PD0332991, Ibrance^®^, Pfizer), were generated. CDK4 and 6 are key regulators in the progression from G1 to S phase, which is a critical checkpoint for DNA replication (7). Palbociclib triggers cell cycle arrest by blocking retinoblastoma (Rb) phosphorylation through binding to the ATP binding site (8) and is approved for the treatment of hormonal receptor-positive, HER2^-^ metastatic BC in combination with anti-hormonal therapy in Europe.

As the VDR is expressed in ±60% of invasive breast carcinomas (9), we postulate that interfering with the vitamin D signaling pathway may be an effective addition to treatment regimens in BC patients. Whereas the combination of palbociclib with hormonally-directed drugs is a first-line treatment in patients with ER^+^/PR^+^ metastatic BC, palbociclib therapy is so far not used for the treatment of TNBC. As TNBC is a highly aggressive subtype of BC, affecting younger women with a high recurrence rate, there is a clear need for the development of better therapies (10, 11).

Therefore, the aim of this study was to elucidate whether combination of a potent vitamin D_3_ analog, such as inecalcitol, with palbociclib treatment, would enhance the antiproliferative effect of both single compounds in different BC subtypes. Furthermore, we wanted to investigate if inecalcitol would increase the sensitivity of TNBC cells to palbociclib treatment. This novel treatment strategy of combining inecalcitol with palbociclib was first investigated on *in vitro* growth of luminal A (ER^+^) BC and compared with the effects observed in basal-like and mesenchymal TNBC cells. In addition, we studied the molecular signaling pathways of this combination therapy in the different cancer types.

Secondly, we evaluated the effect of inecalcitol and palbociclib combination treatment *in vivo* using an ER^+^ and a TNBC cell line-derived xenograft mouse model to evaluate the clinical applicability of this combination treatment on the different BC subtypes.

## 2 Materials and Methods

### 2.1 Cell Lines

All cell lines were authenticated by ATCC *via* short-tandem repeat profiling. The MCF7 (ER^+^) cell line was purchased from ATCC and cultured in Dulbecco’s Modified Eagle’s Medium (DMEM-11880028, Thermo Fisher Scientific) supplemented with 10% fetal bovine serum (FBS), 1% penicillin (10 000 U/mL)-streptomycin (10 000 µg/mL) (P/S, Thermo Fisher Scientific) and 2 mM GlutaMax (35050-038, Thermo Fischer Scientific). T47D (ER^+^) cells were a generous gift from prof. Johan Swinnen, KU Leuven (Leuven, Belgium). Cells were cultured in RPMI 1640 (11835, Thermo Fisher Scientific) containing 2 mM glutamine and supplemented with 10% FBS, 0.5% P/S, D-glucose (2.5 mg/mL) and 0.2 U/mL bovine insulin (Sigma). The BT20, HCC1143 and Hs578T (TNBC) cell lines were a generous gift from prof. Michael J. Duffy, University College Dublin (Dublin, Ireland). These cell lines were cultured and maintained in RPMI 1640 (21875, Thermo Fisher Scientific) containing 2 mM glutamine supplemented with 10% FBS and 0.5% P/S. All cell lines were incubated at 37°C with 5% CO_2_ and passaged *via* trypsinization.

### 2.2 Chemicals

Palbociclib-hydrochloride was obtained from Selleck Chemicals (Houston, TX, USA) and a stock solution of 1 mM was made in phosphate buffered saline (PBS). For *in vivo* use, palbociclib-hydrochloride was dissolved in 50 mM sodium-lactate buffer (pH = 4). Inecalcitol was provided by Hybrigenics SA (Paris, France) and a stock solution of 1 mg/mL was dissolved in ethanol. For *in vivo* experiments, inecalcitol was further diluted in arachis oil.

### 2.3 RNA Extraction and Quantitative Real-Time-PCR Analysis

*VDR, CYP24A1, CYP27B1*, *TRPV6*, *CAT*, *SLC37A2* and *IGFBP3* expression in the different cell lines were determined by qRT-PCR. Total RNA was isolated from cells using the InnuPrep RNA mini kit (Analytikjena) according to the manufacturer’s instructions. Reverse transcription was performed using the SuperScript™ II Reverse Transcriptase (FastGene Scriptase II cDNA kit, Nippon Genetics) from 500 ng RNA and diluted 1/10 before analysis. qPCR reactions were performed with the SYBR Green Mastermix (Life Technologies) or TaqMan Fast Universal PCR master mix (Applied Biosystems, Foster City, CA, USA) in a 7500 Fast sequence detector system (Applied Biosystems). Relative gene expression was calculated with the 2^-ΔΔCt^ method. Gene expression levels were normalized to the expression of *GAPDH*. All qRT-PCR reactions were performed in triplicate. Sequences of primers and probes are summarized in **Table 1**.

**Table 1:** Overview of primer and probe sequences used for qRT-PCR.

Gene name	Gene symbol	*	Sequence
Vitamin D receptor	*VDR*	F R	GGACGCCCACCATAAGACCTA TGGCTCCCTCCACCATCAT
Cytochrome P450 Family 24 Subfamily A Member 1	*CYP24A1*	F R	TATCGCGACTACCGCAAAGA CGGCCAAGACCTCATTGATT
Cytochrome P450 Family 27 Subfamily B Member 1	*CYP27B1*	F R	CCCAGATCCTAACACATTTTGAGG AAAGGGTGATGATGACAGTCTCTTTC
Transient Receptor Potential Cation Channel Subfamily V Member 6	*TRPV6*	F R	GGTTCCTGCGGGTGGAA GAAGGCCTGTGCGTAGCGT
Catalase	*CAT*	F R P	GGTTCCTGCGGGTGGAA GAAGGCCTGTGCGTAGCGT ATCTCAACCGGCAGCGGATCCA
Solute Carrier Family 37 Member 2	*SLC37A2*	F R	GCTGGTCAGTTACACCTTCCTC CCGCCTATGATGCCACCAACAT
Insulin Like Growth Factor Binding Protein 3	*IGFBP3*	F R	CGCTACAAAGTTGACTACGAGTC GTCTTCCATTTCTCTACGGCAGG
Glyceraldehyde-3-Phosphate Dehydrogenase	*GAPDH*	F R	AATCCCATCACCATCTTCCA TGGACTCCACGACGTACTCA

*F, forward primer; R, reverse primer; P, probe.

### 2.4 [^3^H]thymidine Incorporation

BC cells were seeded in 96-well plates and stimulated the next day with a concentration gradient of inecalcitol (10^-6^ M – 10^-13^ M) and/or palbociclib (10^-5^ M – 10^-10^ M) or a single dose of vehicle (EtOH), inecalcitol (10^-8^ M) and/or palbociclib (10^-7^ M). After 72 h of stimulation, 1 µCi [^3^H]thymidine (specific activity of 2 Ci/mmol, PerkinElmer) was added for an additional 4 h incubation period. Thereafter, cells were harvested on filters and incorporated thymidine was counted using a TopCount NXT Scintillation Counter (Packard).

### 2.5 IncuCyte Live-Cell Analysis

BC cells were plated in 96-well plates (TPP) and treated the next day with vehicle (EtOH), inecalcitol (10^-8^ M) and/or palbociclib (10^-7^ M). Cell growth was followed by the IncuCyte Zoom live-cell analysis system for 96 h (BT20-HCC1143 and Hs578T cells) and 120 h (T47D and MCF7 cells). Cell growth was based on confluency rate of the wells and was normalized to the starting cell density between 10-16 h. Difference in cell growth was analyzed by determining the area under the curve (AUC).

### 2.6 DNA Measurement

Cells were seeded into 12-well plates and treated with vehicle (EtOH), inecalcitol (10^-8^ M) and/or palbociclib (10^-7^ M) the next day. After 24, 48 and 72 h of treatment, medium was discarded and cells were washed with PBS. Homogenization buffer was added (0.05 M Na_2_HPO_4_/NaH_2_PO_4_ buffer, 2 M NaCl, 2.10^-3^ M EDTA, pH = 7.4) to each well and the plate was incubated for 1 h at 37°C before scraping and sonication of the cell suspension (ultrasonic processor UP50H, Hielscher; 20 sec, cycle = 1, amplitude = 80%). The amount of DNA was measured by adding 50 µl of the sample to 50 µl of Hoechst solution (1 µg/mL final concentration) (Hoechst 33258-reagens, Calbiochem La Jolla) in a 96-well plate. The plate was read by the fluorescence microplate reader (Synergy H1, Biotek) at excitation wavelength of 360 nm and emission wavelength of 460 nm. Concentration of DNA was determined by using a standard curve based on herring sperm DNA (Boehringer).

### 2.7 Western Blotting

BC cells were seeded in 10 cm dishes and treated the next day with vehicle (EtOH), inecalcitol (10^-8^ M) and/or palbociclib (10^-7^ M) for 72 h. Cells were washed and scraped in ice-cold PBS before lysis in RIPA extraction buffer (50 mM Tris-HCl pH = 8, 150 mM NaCl, 0.1% SDS, 1% NP40, 0.5% Na-deoxycholate diluted in H_2_O, 1x Halt™ protease inhibitor cocktail (Thermo Fisher Scientific), 50 mM NaF, 1 mM Na_3_VO4). Next, protein pellets were sonicated (ultrasonic processor UP50H, Hielscher; cycle = 1, amplitude = 80%) and supernatants were collected after centrifugation (14 000 rpm for 10 min at 4°C). To isolate proteins from tumor tissues, two different isolation protocols were used. First, a small piece of frozen tumor tissue was homogenized in 250 µl RIPA extraction buffer with CK14 beads (1.4 mm zirconium oxide beads, Bertin Technologies) using the Precellys 24 homogenizer (4500 rpm, 2x60 sec) (Avantor). Tissue debris was removed by centrifugation (15 000 rpm for 15 min at 4°C) and supernatant was collected. To remove remaining debris, the samples were additionally sonicated for 5 seconds (ultrasonic processor UP50H, Hielscher; cycle = 1, amplitude = 80%). In the second method, a small piece of frozen tumor tissue was homogenized in 250 µl RIPA extraction buffer with the VDI 12 homogenizer (Avantor). Next, mixed tissues were transferred to Eppendorf tubes and incubated for 1 h rotating at 4°C. Tissue debris was removed by centrifugation (15 000 rpm for 30 min at 4°C) and supernatant was collected. Protein concentration was determined based on the BCA method (Pierce™ BCA Protein Assay Kit, Thermo Fisher Scientific) according to the manufacturer’s recommendations. Total cell proteins (20 μg) were separated by 4-12% Bis-Tris SDS-PAGE (Thermo Fisher Scientific) or by Invitrogen NuPAGE 3-8% Tris-acetate gels for high molecular weight proteins (Thermo Fisher Scientific) and transferred to nitrocellulose membranes (Amersham™ Protran^®^ Premium Western blotting membranes, nitrocellulose, GE Healthcare). Membranes were blocked with 5% bovine serum albumin (Fisher Scientific) or 5% dry milk dissolved in 0.1% Tween20 in Tris-Buffered Saline (TBS) for 60 min and overnight incubated with the primary antibody (**Table 2**) at 4°C. Next, after washing, membranes were incubated for 1 h at room temperature with the peroxidase conjugated secondary antibody (**Table 2**). Protein bands were visualized using enhanced chemiluminescence as described by the supplier (Western lightning ECL PRO, PerkinElmer) and quantified by laser densitometric scanning (Avantor GelDoc analysis).

**Table 2:** Overview of antibodies used for western blot and immunofluorescence.

	Protein	Host	Label	Dilution	Supplier	Catalog number
Primary antibodies	Rb	Rabbit	–	1/1000	CST	9313
	p-Rb (Ser807/811)	Rabbit	–	1/1000	CST	9308
	CDK2	Rabbit	–	1/1000	CST	2546
	p-CDK2 (Thr160)	Rabbit	–	1/1000 (*in vivo*: 1/500)	CST	2561
	Cyclin E1	Mouse	–	1/1000	CST	4129
	VDR (D2K6W)	Rabbit	–	1/500	CST	12550
	PARP	Rabbit	–	1/1000	CST	9542
	mTOR	Rabbit	–	1/1000	CST	2972
	p-mTOR (Ser2448)	Rabbit	–	1/1000	CST	2971
	Akt	Rabbit	–	1/1000	CST	9272
	p-Akt (Ser473)	Rabbit	–	1/1000	CST	9271
	P70S6K	Rabbit	–	1/1000	CST	2708
	p-P70S6K (Ser371)	Rabbit	–	1/1000	CST	9208
	p-P70S6K (Thr389)	Rabbit		1/1000	CST	9234
	S6RP	Rabbit	–	1/1000	CST	2217
	p-S6RP (Ser235/236)	Rabbit	–	1/1000	CST	4856
	4E-BP1	Rabbit	–	1/1000	CST	9452
	p-4E-BP1 (Thr37/46)	Rabbit	–	1/1000	CST	9549
	β-actin	Mouse	–	1/5000	Sigma Aldrich	A5441
	vinculin	Rabbit	–	1/1000	CST	4650
	Ki67	Rabbit	–	1/5000	Novocastra	NCLKi67p
Secondary antibodies	Anti-mouse	Rabbit	HRP	1/5000	DAKO	P0161
	Anti-rabbit	Goat	HRP	1/5000 or 1/500 (immuno)	DAKO	P0448

### 2.8 Flow Cytometry

Cells were seeded in 6-well plates and treated during 72 h with vehicle (EtOH), inecalcitol (10^-8^ M) and/or palbociclib (10^-7^ M). For cell cycle analysis, cells (10^6^) were fixed with 70% EtOH, washed and permeabilized in 0.1% Tween20 in PBS before staining with 0.5 mg/mL propidium iodide (PI) containing 1 mg/mL RNase A. Mitochondrial ROS levels were measured by incubating the cells for 10 min at 37°C with 2.5 µM of the fluorescent probe dye MitoSOX Red (Thermo Fischer Scientific). Mitochondria were stained using 200 nM MitoTracker Deep Red fluorescent probe (Thermo Fischer Scientific) for 30 min at 37°C. Apoptosis was detected using the FITC Annexin V apoptosis Detection kit I (BD Pharmingen), following the instructions of the manufacturer. Unstained and single stained controls were included. Viable cells were analyzed as PI and Annexin V negative. Apoptotic cells were analyzed as Annexin V positive. FACS samples were prepared in duplicates. Fluorescence was determined on the Gallios flow cytometer (Beckman Coulter, FACS core KU Leuven) or on the BD FACSCanto II flow cytometer (FACS core KU Leuven) and data were analyzed with the Kaluza software or Flowing software 2.

### 2.9 Immunofluorescence

Tumor tissues were fixed in 2% paraformaldehyde (PFA), subsequently dehydrated, embedded in paraffin and sectioned at 4 µm. After deparaffinization, slides were washed with AD before antigen retrieval at pH = 9 for 15 min in a steamer (Target retrieval solution, DAKO, S1700). Next, slides were washed with PBS and 3% H_2_O_2_ in PBS for 10 min. Before 45 min blocking in TNB buffer [0.1 M Tris-HCl pH = 7.5; 0.15 M NaCl; 0.5% blocking reagent (FP1012, TSA-kit, Perkin Elmer, NEL701A001KT)], slides were washed with AD, PBS and Tris-buffered saline (TBS) (2 x 5 min), followed by overnight incubation with Ki67 primary antibody at 4°C in TNB (**Table 2**). The next day, slides were washed with TBS before incubation with the secondary antibody in TNB for 1 h at room temperature (**Table 2**). Next, samples were washed with TBS before 10 min incubation in fluorescein-tyramide (FITC) (TSA-kit, Perkin Elmer, NEL701A001KT) (1/50 in amplification diluent). After washing with TBS, tissue sections were stained with 1 µg/mL Hoechst for 10 min (Sigma), before washing in TBS and mounting with fluomount (Agilent). Images of the stainings were taken using the Zeiss Axio Imager.M2 microscope and analyzed with ImageJ software.

### 2.10 *In Vivo* Xenograft Model

The BC xenograft model was generated by injecting 6-7 week old female NMRI nude mice (Janvier) subcutaneously in each flank with MCF7 or BT20 cells (5.10^6^) that were resuspended in Matrigel (356237, Corning). When tumors reached an average volume of 400-500 mm^3^, they were transplanted into new NMRI nude mice (Janvier) to generate a xenograft model (F1). All experiments were performed on xenograft F2 mice. When tumors reached an average volume of 100 mm^3^, mice were stratified into four treatment groups: (1) vehicle group [arachis oil and sodium lactate buffer (50 mM, pH = 4)]; (2) inecalcitol (25 µg/kg) daily; (3) palbociclib (50 mg/kg) once every two days and (4) combination group. Treatments were given through oral gavage 6 times a week until the end of the experiment (49 days for MCF7 and 28 days for BT20 xenograft model). Tumor volumes were determined at least once a week using a caliper and calculated by the formula (ab^2^)/2 (a: length and b: width of the tumor). At least 8 mice per experimental group were analyzed with a tumor on each flank. At the end of the experiment, mice were put into metabolic cages for 24 h to analyze food intake and to collect urine. Serum was collected through cardiac puncture. Bone, kidney and intestine were dissected and either fixed in 2% PFA (bone) or snap frozen in liquid N_2_ (kidney and intestine) to study calcemic side effects. Tumors were dissected and divided in different parts for protein and immunohistochemical analysis. All animal experiments were approved by the ethical committee of the KU Leuven (P200/2017).

### 2.11 Statistical Analysis

Statistical analysis was performed with Prism 9.1.0 (GraphPad Software, La Jolla CA, USA). All data were expressed as mean ± standard of error of the mean (SEM) unless otherwise stated. Differences between groups were analyzed using t-test, one-way ANOVA or Mixed-effects analysis, where appropriate, followed by Sidak’s multiple comparison test. Differences were significant for p ≤ 0.05(*), p ≤ 0.01(**), p ≤ 0.001(***) and p ≤ 0.0001(****).

## 3 Results

### 3.1 VDR is Expressed and Activated in all Investigated BC Cell Lines

The major aim of our study was to investigate the anticancer activity of the combination of inecalcitol, a potent vitamin D_3_ analog, and the CDKI palbociclib in different BC models. As BC can be subdivided into different subgroups, two ER^+^ BC cell lines (T47D and MCF7) and three cell lines derived from two different TNBC subtypes (basal-like; BT20 and HCC1143 – mesenchymal; Hs578T cells) were investigated.

**Figure 1:**
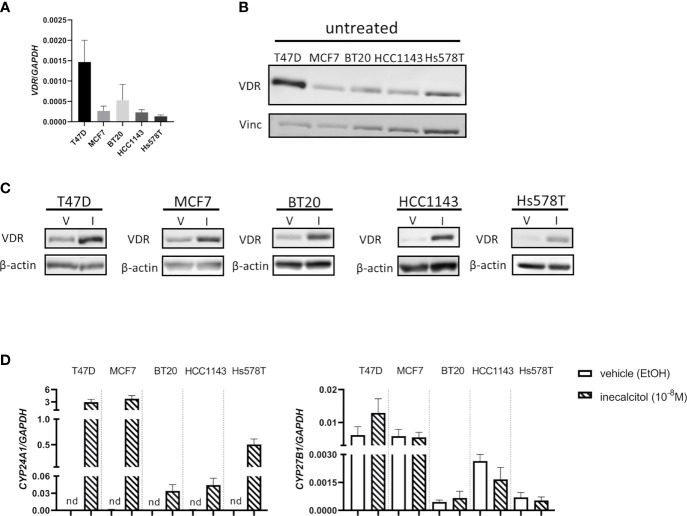
VDR expression in the different BC cell lines. **(A)** *VDR* mRNA expression levels in T47D, MCF7, BT20, HCC1143 and Hs578T cells. Levels were normalized for *GAPDH* expression (n=3 performed in duplicate) **(B)** Basal VDR protein (49 kDa) expression in T47D, MCF7, BT20, HCC1143 and Hs578T cells. Vinculin (124 kDa) was used as loading control. **(C)** VDR protein (49 kDa) expression in the different cell lines after treatment with vehicle (V) (EtOH) or inecalcitol (I) (10^-8^ M, 72 h). β-actin (42 kDa) was used as loading control. **(D)** *CYP24A1* and *CYP27B1* expression levels in T47D, MCF7, BT20, HCC1143 and Hs578T cells after treatment with vehicle or inecalcitol (10^-8^ M, 72 h). Levels were normalized for *GAPDH* expression (n=2-5 performed in duplicate), nd: not detected.

As the VDR mediates the antiproliferative effects of 1,25(OH)_2_D_3_ and its analogs, we first determined VDR expression in all investigated BC cell lines, both at mRNA and protein level. All BC cell lines express the VDR at mRNA and protein level (**Figures 1A, B**) and VDR protein levels were induced after a 72 h incubation period with inecalcitol (10^-8^ M) (**Figure 1C**). To further evaluate the activation of VDR signaling after inecalcitol treatment, the expression of *CYP24A1*, responsible for catabolizing 1,25(OH)_2_D_3_ and among the most sensitive VDR-target genes, and *CYP27B1*, encoding for the activating enzyme 1α-hydroxylase, was investigated. Relative *CYP27B1* expression was not altered upon treatment with inecalcitol (10^-8^ M, 72 h) in any of the BC cell lines, whereas the expression of *CYP24A1* was induced in all BC cell lines (**Figure 1D**). Highest upregulation of *CYP24A1* levels was detected in ER^+^ T47D and MCF7 cells. Furthermore, the expression of additional VDR target genes, known to be regulated by 1,25(OH)_2_D_3_ or inecalcitol in BC cells (12, 13), was analyzed in the different BC cell lines after treatment with inecalcitol (10^-8^ M, 72 h) (**Supplementary Figure 1**). Relative expression of transient receptor potential cation channel subfamily V member 6 (*TRPV6*) was significantly upregulated by inecalcitol in all BC cell lines, while expression of catalase (*CAT*), solute carrier family 37 member 2 (*SLC37A2*), and insulin like growth factor binding protein 3 (*IGFBP3*) was induced in most but not all BC cell lines. In conclusion, these data illustrate that ER^+^ and TNBC cells express and activate VDR upon inecalcitol stimulation, but also that the VDR-induced signaling cascades are not identical in all BC cell lines.

### 3.2 Combined Treatment of Inecalcitol With Palbociclib Reduces Cell Proliferation and Cell Growth in BC Cells

**Figure 2:**
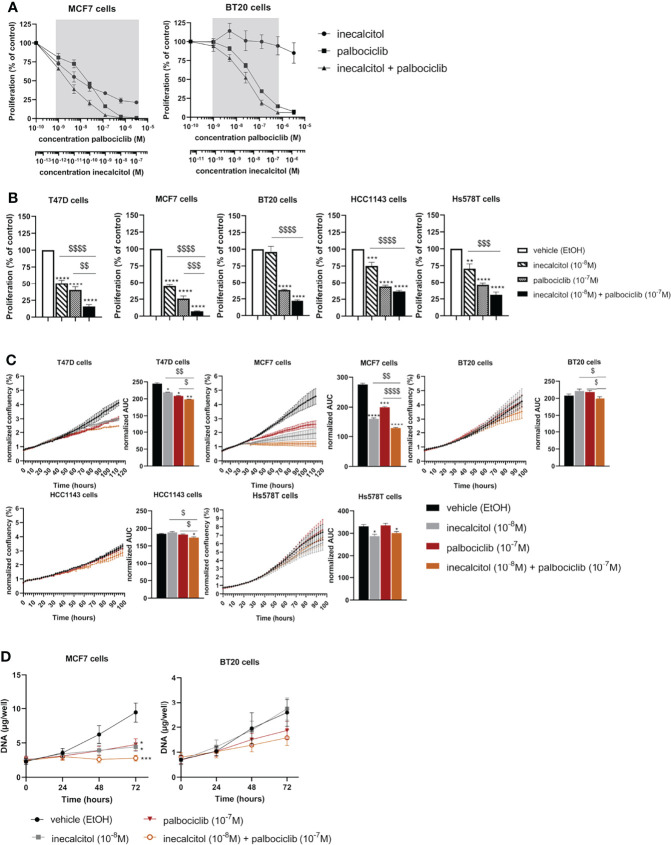
Effect of inecalcitol and/or palbociclib treatment on BC cell growth and proliferation. **(A)** Dose titration analysis of inecalcitol and palbociclib alone or combined in MCF7 and BT20 cells. CI < 1; synergistic effect, in the zone indicated in grey (n=2-4 with 10 technical replicates) **(B)** Combined treatment of inecalcitol (10^-8^ M) and palbociclib (10^-7^ M) decreased cell proliferation in T47D cells (84%), MCF7 cells (93%), BT20 cells (78%), HCC1143 cells (63%), and Hs578T cells (69%) compared to vehicle treatment after 72 h (n = 4-5 with 5-10 technical replicates). Data were expressed relative to vehicle (%). **(C)** Cell growth was analyzed with IncuCyte live cell analysis for T47D, MCF7, BT20, HCC1143 and Hs578T cells. Well confluency was normalized to start density between 10-16 h. Differences between AUC were analyzed by multiple unpaired t-testing (n = 2-6 with 5 technical replicates). **(D)** Cell growth was assessed by DNA staining in MCF7 and BT20 cells after 24, 48 and 72 h of treatment (n = 4-5 performed in duplicate). Data shown as mean ± SEM. *p ≤ 0.05; **p ≤ 0.01; ***p ≤ 0.001; ****p ≤ 0.0001 compared to vehicle; ^$^p ≤ 0.05; ^$$^p ≤ 0.01; ^$$$^p ≤0.001; ^$$$$^p ≤ 0.0001 compared to inecalcitol or palbociclib monotherapy (one-way ANOVA, Sidak’s multiple comparison test).

To define the optimal combination dose for inecalcitol and palbociclib combination therapy, we selected one ER^+^ BC (MCF7) cell line and one TNBC (BT20) cell line to perform a dose titration study. Based on the principle of Chou-Talalay, we identified that a combination of 10^-8^ M inecalcitol with 10^-7^ M palbociclib resulted in a synergistic growth inhibition in both cell lines as demonstrated by reduced [^3^H]thymidine incorporation after 72 h of treatment [combination index (CI)<1 (indicated by the grey zones) (calculated with CompuSyn software) (14) (**Figure 2A**). Next, we investigated the antiproliferative effect of this combination therapy on the different BC cell lines. Cells were treated during 72 h with vehicle (EtOH), inecalcitol, palbociclib or a combination of both. The combination therapy decreased cell proliferation significantly compared to vehicle, inecalcitol and palbociclib treatment in ER^+^ T47D and MCF7 cells (**Figure 2B**). However, in all investigated TNBC cell lines, [^3^H]thymidine incorporation in the combination-treated cells was significantly lower than in vehicle- and inecalcitol-treated cells, but not than in palbociclib-treated cells (**Figure 2B**), indicating that ER^+^ cell lines (T47D and MCF7) were more responsive to the antiproliferative effect of the combination therapy than TNBC cell lines.

We further analyzed the effect of the combination therapy on cell growth by performing IncuCyte live-cell analysis on all BC cell lines (**Figure 2C**). Cell growth, based on confluency rate, was significantly decreased after inecalcitol and palbociclib combination treatment compared to vehicle, inecalcitol or palbociclib monotherapy for T47D (ER^+^), MCF7 (ER^+^) and HCC1143 (TNBC) cells (**Figure 2C**). Especially the cell growth of MCF7 (ER^+^) cells was nearly completely inhibited after treatment with inecalcitol in combination with palbociclib. For BT20 (TNBC) cells, the combination therapy decreased cell growth significantly compared to inecalcitol or palbociclib monotherapy. For Hs578T (TNBC) cells, inecalcitol treatment decreased cell growth significantly although there was no additional effect of palbocilib (**Figure 2C**). Again, treatment with the combination therapy decreased cell growth more efficiently in ER^+^ cell lines than in TNBC cells.

To further evaluate the effect of the combination therapy on cell growth, we chose the most responsive ER^+^ BC cell line - MCF7 - and the most sensitive TNBC cell line -BT20 - for further analysis. DNA levels were measured after 24, 48 and 72 h of treatment with inecalcitol and palbociclib alone or combined. For MCF7 (ER^+^) cells, the amount of DNA was significantly decreased after 72 h of inecalcitol and palbociclib treatment alone (**Figure 2D**). The combination treatment of inecalcitol and palbociclib further reduced DNA levels compared to both mono treatments, although not significantly (**Figure 2D**). For BT20 (TNBC) cells, the amount of DNA was decreased after treatment with the combination therapy but this effect was not significant (**Figure 2D**). In summary, the different cell proliferation assays indicated that the combination therapy decreased cell proliferation and growth in all BC cells, but the effect was more pronounced in ER^+^ BC cells than in TNBC cells.

### 3.3 Treatment With Inecalcitol and Palbociclib Blocks Cell Cycle Progression by Downregulating Cell Cycle Regulatory Proteins

To further study the effect of the combination therapy on cell proliferation, we performed cell cycle analysis.

**Figure 3:**
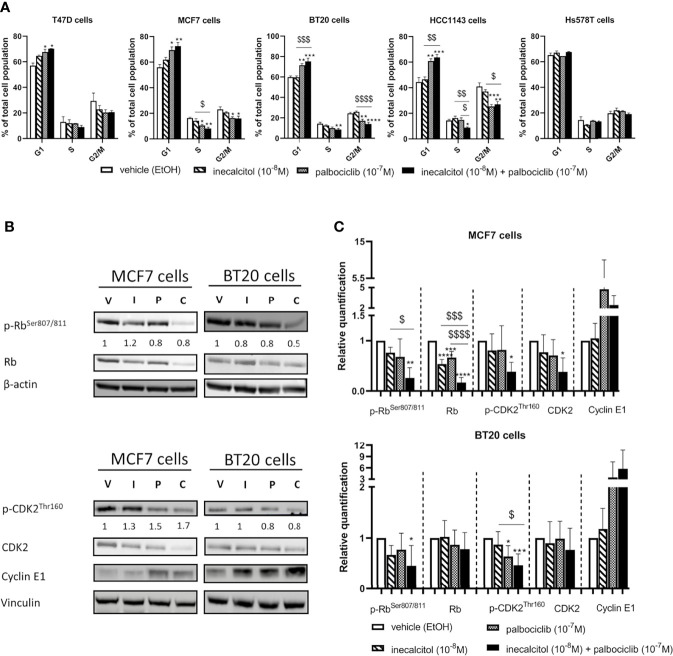
Effect of inecalcitol and/or palbociclib treatment on cell cycle regulation. **(A)** PI FACS analysis showed the number of cells in the different phases of the cell cycle in all investigated cell lines after treatment with vehicle (EtOH, 72 h), inecalcitol (10^-8^ M, 72 h), palbociclib (10^-7^ M, 72 h) or the combination of both (72 h) (n=2-5 performed in duplicate). Data shown as mean ± SEM. **(B)** Protein expression of (p)-Rb (110 kDa), (p)-CDK2 (33 kDa) and Cyclin E1 (48 kDa) in MCF7 and BT20 cells after 72 h treatment (one representative blot shown). The ratio of phosphorylated to non-phosphorylated relative protein expression is indicated under the representative blot. Data are expressed as fold increase vs control (control value = 1). **(C)** Relative quantification of protein levels of (p)-Rb, (p)-CDK2 and Cyclin E1 in MCF7 and BT20 cells (n=4-6). β-actin (42 kDa) and vinculin (124 kDa) were used as loading control. V = vehicle (EtOH), I = inecacitol (10^-8^ M), P = palbociclib (10^-7^ M) and C = combination of inecalcitol (10^-8^ M) and palbociclib (10^-7^ M). Data shown as mean ± SD. *p ≤ 0.05; **p ≤ 0.01; ***p ≤ 0.001; ****p ≤ 0.0001 compared to vehicle; ^$^p ≤ 0.05; ^$$^p ≤ 0.01; ^$$$^p ≤ 0.001; ^$$$$^p ≤ 0.0001 compared to inecalcitol or palbociclib monotherapy (one-way ANOVA, Sidak’s multiple comparison test).

In all investigated BC cell lines, except for Hs578T cells, the combination therapy significantly increased the number of cells in the G1 phase compared to vehicle treatment (**Figure 3A**). In BT20 (TNBC) and HCC11143 (TNBC) cells, this increase in G1 cells was significant compared to inecalcitol mono treatment. The number of S phase cells was significantly decreased after treatment with the combination therapy compared to vehicle treatment in MCF7 (ER^+^), BT20 (TNBC) and HCC1143 (TNBC) cells. More specific, in MCF7 (ER^+^) and HCC1143 (TNBC) cells, the combination treatment reduced the number of S phase cells significant compared to inecalcitol mono treatment, while in HCC1143 (TNBC) cells there was a significant decrease in S phase cells compared to palbociclib monotherapy. Also, palbociclib treatment alone or combined with inecalcitol significantly reduced the number of cells in G2/M phase compared to vehicle treatment in MCF7 (ER^+^), BT20 (TNBC) and HCC1143 (TNBC) cells (**Figure 3A**).

As we observed changes in cell cycle regulation after treatment with the combination therapy, we analyzed the expression of different important cell cycle regulatory proteins such as retinoblastoma (Rb), cyclin-dependent kinase 2 (CDK2) and Cyclin E1. We selected one ER^+^ BC cell line - MCF7- and one TNBC cell line -BT20- for further analysis of the signaling pathways responsible for the antiproliferative effects. In MCF7 (ER^+^) cells, both total and phosphorylated (p) levels of Rb were significantly downregulated after 72 h of treatment with the combination therapy compared to vehicle treatment. Moreover, after the combination treatment both total and p-Rb levels were significantly lower than after inecalcitol monotherapy, while total Rb levels were also significantly lower than after palbociclib monotherapy (**Figures 3B, C**). In addition, both total and phosphorylated levels of CDK2 were decreased upon treatment with the combination therapy compared to vehicle treatment. As both total and phosphorylated protein levels were reduced, no major alterations were observed in the ratio of phosphorylated to non-phosphorylated Rb and CDK2 protein levels. (**Figures 3B, C).** In contrast, Cyclin E1 levels were upregulated, mainly after palbociclib treatment, although not significantly (**Figures 3B, C**). In the TNBC BT20 cell line, combination therapy decreased p-Rb levels significantly compared to vehicle treatment, whereas p-CDK2 levels were significantly reduced compared to vehicle and inecalcitol treatment. No effect was observed on total levels of Rb and CDK2 (**Figures 3B, C**). For both Rb and CDK2, the ratio of phosphorylated to non-phosphorylated protein levels was slightly decreased upon treatment with the combination therapy (**Figure 3B**). Again, Cyclin E1 levels were upregulated, mainly after treatment with the combination therapy, although not significantly (**Figures 3B, C**). These results demonstrate that the combination treatment with inecalcitol and palbociclib strongly suppressed protein levels of cell cycle regulatory proteins in ER^+^ (MCF7) cells, while only a mild down-regulation was observed in TNBC (BT20) cells.

### 3.4 Effect of Inecalcitol and Palbociclib Treatment on the PI3K/Akt/mTOR Pathway

As we observed a decreased cell growth and proliferation in all the BC cell lines after treatment with the combination therapy, we further wanted to evaluate the effect of this combination therapy on the PI3K/Akt/mTOR pathway, which is an important regulator of cell growth and proliferation.

**Figure 4:**
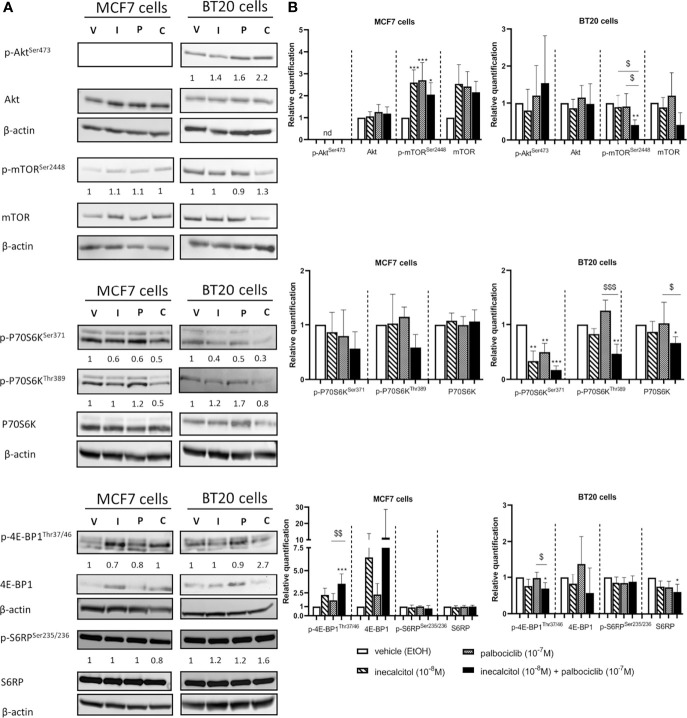
Effect of inecalcitol and/or palbociclib treatment on PI3K/Akt/mTOR pathway. **(A)** Protein expression of (p)-Akt (60 kDa), (p)-mTOR (289 kDa), (p)-P70S6K (70-85 kDa), (p)-4E-BP1 (15-20 kDa), (p)-S6RP (32 kDa) in MCF7 and BT20 cells after 72 h of treatment (one representative blot is shown). The ratio of phosphorylated to non-phosphorylated relative protein expression is indicated under the representative blot. Data are expressed as fold increase vs control (control value = 1). **(B)** Relative quantification of protein levels of (p)-Akt, (p)-mTOR, (p)-P70S6K, (p)-4E-BP1, (p)-S6RP in MCF7 and BT20 cells (n= 3-7). β-actin (42 kDa) was used as loading control. V = vehicle (EtOH), I = inecacitol (10^-8^ M), P = palbociclib (10^-7^ M) and C = combination of inecalcitol (10^-8^ M) and palbociclib (10^-7^ M). Data shown as mean ± SD. ndthe not detected. *p ≤ 0.05; **p ≤ 0.01; ***p ≤ 0.001 compared to vehicle; ^$^p ≤ 0.05; ^$$^p ≤ 0.01; ^$$$^p ≤ 0.001 compared to inecalcitol or palbociclib monotherapy (one-way ANOVA, Sidak’s multiple comparison test).

We first looked at the protein expression of total and phosphorylated Akt, yet, in MCF7 cells p-Akt^Ser473^ could not be detected. Treatment of the MCF7 (ER^+^) and BT20 (TNBC) cells with inecalcitol and palbociclib alone or combined did not affect the expression of total and p-Akt levels. However, in MCF7 (ER^+^) cells, total and phosphorylated levels of mammalian target of rapamycin (mTOR) were upregulated after mono and combined treatment, while opposite effects were seen in BT20 (TNBC) cells. Indeed, in BT20 (TNBC) cells the combination treatment decreased the expression of p-mTOR compared to vehicle and mono treatments (**Figures 4A, B**). However, in both cell lines, the ratio of p-mTOR/mTOR protein levels was not altered. Next, we evaluated the expression of downstream targets of mTOR such as P70S6K (the 70-kDa ribosomal protein S6 kinase), and its downstream target S6 ribosomal protein (S6RP) and 4E-BP1 (eukaryotic translation initiation factor 4E (eIF4E)-binding protein 1), all involved in the regulation/initiation of protein synthesis and/or protein translation important for cell growth. In MCF7 (ER^+^) cells, no significant changes were detected in total and p-P70S6K -both at serine and threonine phosphorylation site- or in total and p-S6RP. However, we observed a reduction in the ratio of phosphorylated to total P70S6K protein levels after treatment with the combination therapy at both phosphorylation sites. Yet, p-4E-BP1 levels were strongly upregulated after treatment with the combination therapy compared to vehicle and palbociclib treatment, but the ratio of p-4E-BP1/4E-BP1 protein levels was not altered (**Figures 4A, B**). In contrast, in BT20 (TNBC) cells the mono and combination treatments decreased the expression of p-P70S6K^Ser371^ compared to vehicle treatment, while p-P70S6K^Thr389^ was only decreased after combination therapy. In total S6RP protein levels only a slight reduction was observed after the combination treatment. Also, p-4E-BP1 protein levels were slightly, but significantly reduced after the combination treatment (**Figures 4A, B**). In summary, these findings indicate that PI3K/Akt/mTOR pathway is differently regulated in MCF7 (ER^+^) cells than in BT20 (TNBC) cells.

### 3.5 Treatment With Inecalcitol and Palbociclib Induces Apoptosis in ER^+^ MCF7 but not in TNBC BT20 Cells

We next questioned whether also cell survival was affected by inecalcitol and/or palbociclib treatment.

**Figure 5:**
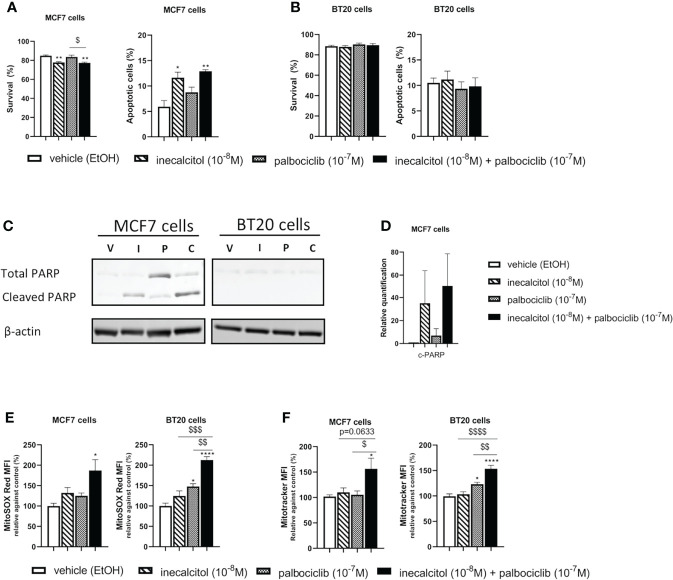
Effect of inecalcitol and/or palbociclib treatment on apoptosis and mitochondrial homeostasis **(A)** Cell survival and apoptosis were determined by AnnexinV-PI FACS analysis in MCF7 cells after treatment with vehicle (EtOH, 72 h), inecalcitol (10^-8^ M, 72 h), palbociclib (10^-7^ M, 72 h) or the combination of both (72 h) (n=3 performed in duplicate). Data shown as mean ± SEM. **(B)** Cell survival and apoptosis were determined by AnnexinV-PI FACS analysis in BT20 cells after treatment with vehicle (EtOH, 72 h), inecalcitol (10^-8^ M, 72 h), palbociclib (10^-7^ M, 72 h) or the combination of both (72 h) (n=3 performed in duplicate). Data shown as mean ± SEM. **(C)** PARP cleavage (89 kDa) in MCF7 and BT20 cells after treatment with vehicle (EtOH, 72 h), inecalcitol (10^-8^ M, 72 h), palbociclib (10^-7^ M, 72 h) or the combination of both (72 h). β-actin (42 kDa) was used as loading control. One representative blot is shown. **(D)** Relative quantification of cleaved PARP in MCF7 cells after 72 h of treatment (n=3). V = vehicle (EtOH), I = inecacitol (10^-8^ M), P = palbociclib (10^-7^ M) and C = combination of inecalcitol (10^-8^ M) and palbociclib (10^-7^ M). Data shown as mean ± SD. **(E)** Mitochondrial ROS levels were determined in MCF7 and BT20 cells after treatment with vehicle (EtOH, 72 h), inecalcitol (10^-8^ M, 72 h), palbociclib (10^-7^ M, 72 h) or the combination of both (72 h) (n=3 performed in duplicate). Data were expressed relative to vehicle (%) and shown as mean ± SEM. **(F)** Mitotracker Deep Red was analyzed in MCF7 and BT20 cells after treatment with vehicle (EtOH, 72h), inecalcitol (10^-8^ M, 72 h), palbociclib (10^-7^ M, 72 h) or the combination of both (72 h) (n=5/6 performed in duplicate). Data were expressed relative to vehicle (%) and shown as mean ± SEM. *p ≤ 0.05; **p ≤ 0.01; ****p ≤ 0.0001 compared to vehicle; ^$^p ≤ 0.05; ^$$^p ≤ 0.01; ^$$$^p ≤ 0.001; ^$$$$^p ≤ 0.0001 compared to inecalcitol or palbociclib monotherapy (one-way ANOVA, Sidak’s multiple comparison test).

Therefore, we performed Annexin V-PI FACS analysis. In ER^+^ MCF7 cells, inecalcitol treatment significantly reduced cell survival and increased the number of apoptotic cells compared to vehicle treatment (**Figure 5A**). No additional effect of palbociclib treatment was observed on cell survival or apoptosis. In TNBC BT20 cells, there was no effect on cell survival or apoptosis after mono or combination therapy (**Figure 5B**). Analysis of total and cleaved PARP expression showed PARP cleavage after treatment with inecalcitol and the combination therapy in MCF7 cells, while almost no PARP expression was detected in BT20 cells (**Figures 5C, D**).

As apoptosis can be triggered or induced by production of reactive oxygen species (ROS), we investigated the levels of mitochondrial ROS after treatment with mono or combination therapy. In both BC cell lines, mitochondrial ROS levels were significantly increased after 72 h of combination treatment with inecalcitol and palbociclib compared to vehicle treatment (**Figure 5E**). In TNBC BT20 cells, the increase in mitochondrial ROS levels was also significant compared to inecalcitol and palbociclib monotherapy (**Figure 5E**).

As we observed changes in mitochondrial ROS levels, we further evaluated mitochondrial homeostasis by performing Mitotracker Deep Red staining to determine mitochondrial content after 72 h of treatment. In both cell lines, Mitotracker staining was upregulated after treatment with the combination therapy compared to vehicle and palbociclib treatment (**Figure 5F**). For TNBC BT20 cells, Mitotracker staining was also significantly increased compared to inecalcitol treatment (**Figure 5F**).

In summary, the combination therapy not only affected cell proliferation in ER^+^ MCF7 BC cells, also cell survival was decreased. However, no effect was observed in TNBC BT20 cells. Interestingly, the combination therapy alters mitochondrial homeostasis by upregulation of mitochondrial ROS and content in both cell lines.

### 3.6 Evaluation of the Inecalcitol and Palbociclib Combination Therapy *In Vivo* Using a Cell-Line Derived Xenograft Mouse Model

The combined treatment of inecalcitol and palbociclib not only induced programmed cell death in ER^+^ MCF7 cells, but also almost completely blocked cell proliferation. Therefore, the potency of this promising treatment strategy was evaluated in a MCF7 xenograft model and compared with the anticancer effects of this combination treatment in TNBC BT20 xenografts.

**Figure 6:**
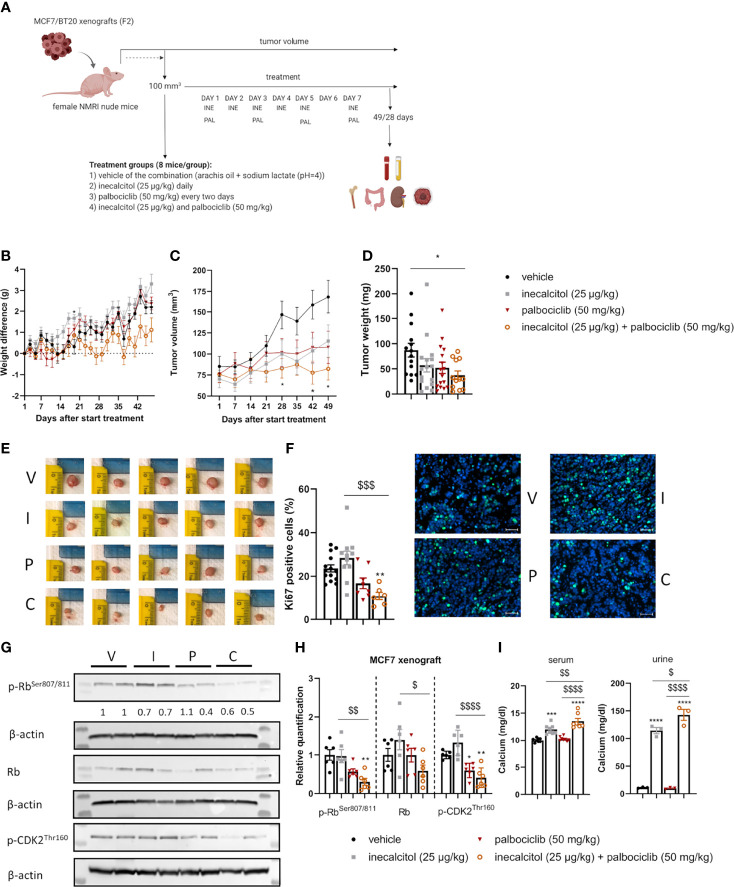
Analysis of the *in vivo* effect of inecalcitol and palbociclib treatment in an ER^+^ MCF7 xenograft model. **(A)** Experimental setup of *in vivo* experiments. Created with BioRender.com. **(B)** Weight difference compared to start weight during treatment (Mixed effect analysis followed by one-way ANOVA, Sidak’s multiple comparison test). **(C)** Tumor volume during treatment (8 mice/group) (Mixed effect analysis followed by one-way ANOVA, Sidak’s multiple comparison test). **(D)** Tumor weight at day 49 of treatment (12-16 tumors/group). **(E)** Photographs of tumors isolated at day 49 from the different treatment groups: vehicle (V), inecalcitol (25 µg/kg) (I), palbociclib (50 mg/kg) (P) and combination (C). **(F)** Immunohistochemistry analysis of Ki67 staining on tumor samples (6-14 tumors/group) and representative pictures of Ki67 staining (green) with DAPI counter staining (blue) per group: vehicle (V), inecalcitol (25 µg/kg) **(I)**, palbociclib (50 mg/kg) (P) and combination (C). Scale bar = 50 µm. **(G)** P-Rb, total Rb (110 kDa) and p-CDK2 (33 kDa) protein expression in tumors from vehicle (V), inecalcitol (25 µg/kg) **(I)**, palbociclib (50 mg/kg) (P) and combination (C) treated mice (6 tumors/group). One representative blot is shown. β-actin (42 kDa) was used as loading control. The ratio of phosphorylated to non-phosphorylated relative protein expression is indicated under the representative blot. Data are expressed as fold increase vs control (control value = 1). **(H)** Relative quantification of p-Rb, total Rb and p-CDK2 protein expression (n=6 tumors/group). Data shown as mean ± SD. **(I)** Calcium analysis of serum (7-8 mice/group) and urine (3 pooled samples/group). Data shown as mean ± SEM of one experiment (8 mice/group). *p ≤ 0.05; **p ≤ 0.01; ***p ≤ 0.001; ****p ≤ 0.0001 compared to vehicle; ^$^p ≤ 0.05; ^$$^p ≤ 0.01; ^$$$^p ≤0.001; ^$$$$^p ≤ 0.0001 compared to inecalcitol or palbociclib monotherapy (one-way ANOVA, Sidak’s multiple comparison test).

Single and combination dose-titration studies were performed for inecalcitol and palbociclib to determine the optimal treatment dose. In these titration studies, mice were orally treated for two weeks with inecalcitol (25 or 40 µg/kg every day), palbociclib (50 mg/kg every two days or 75 mg/kg every three days) or the combination of both (**Supplementary Figure 2A**). Weight was evaluated daily during treatment and at the end of the experiment serum and urine were collected to investigate calcemic side effects. Weight was not changed during treatment (data not shown). Inecalcitol or palbociclib mono treatment did not increase serum calcium levels. Only a slight increase in serum and urine calcium levels was observed in the combination therapy but this was not hypercalcemic for the combination dose of 25 µg/kg/d for inecalcitol and 50 mg/kg/2d for palbociclib (**Supplementary Figure 2B**). Based on this pilot experiment, this combination dose was selected for further experiments and treatment was given as indicated in **Figure 6A**.

#### 3.6.1 Low Dose Combination of Inecalcitol and Palbociclib Treatment Efficiently Decreased Tumor Growth in an ER^+^ MCF7 Xenograft Model

We first evaluated the combination therapy in a xenograft model derived from ER^+^ MCF7 cells (**Figure 6A**). Tumor growth was assessed for 49 days. Both mono and combined treatment did not induce weight loss during treatment (**Figure 6B**). Interestingly, the combination therapy almost completely prevented tumor growth and at the end of the experiment, tumor volume in the combination group was 48.8% of the tumor volume of vehicle treated mice (p<0.05, **Figure 6C**). In addition, tumor weight was significantly decreased with 56% after combination compared to vehicle treatment, confirming the anticancer effects of the combination treatment (**Figures 6D, E**). Also, histochemical analysis of Ki67-positive tumor cells illustrated the significant decrease in cell proliferation after treatment with the combination therapy (54% compared to vehicle) (**Figure 6F**). Protein analysis showed a significantly reduced p-Rb and p-CDK2 expression in ER^+^ MCF7 xenografts after the combination therapy compared to vehicle and inecalcitol treatment. Moreover, the ratio of p-Rb/Rb protein levels was decreased after treatment with the combination therapy. (**Figures 6G, H**). In contrast to the pilot studies, we observed a significant increase in serum and urine calcium concentrations after monotherapy with inecalcitol and after the combination treatment and this increase was slightly more pronounced after the combination treatment (**Figure 6I**). However, bone calcium content was not altered after either treatment regimen (**Supplementary Figure 3A**).

#### 3.6.2 Combination of Inecalcitol and Palbociclib Treatment Did Not Decrease Tumor Growth in a TNBC BT20 Xenograft Model

**Figure 7:**
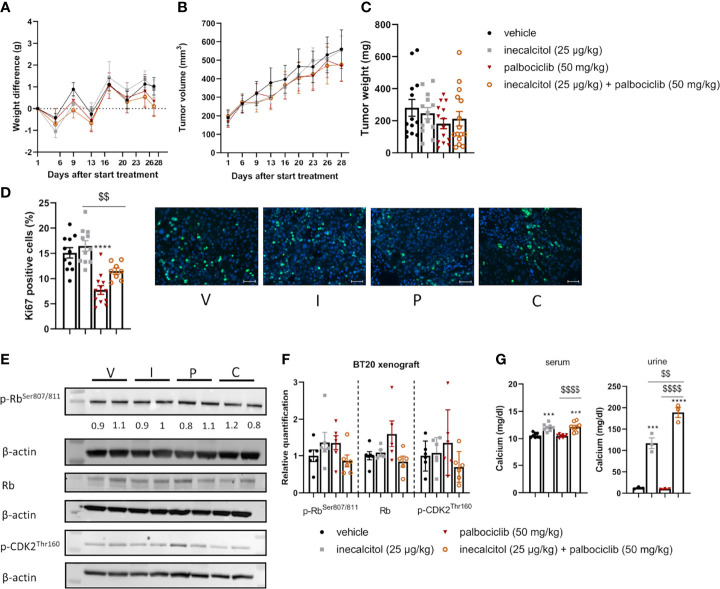
Analysis of the *in vivo* effect of inecalcitol and palbociclib treatment in a TNBC BT20 xenograft model. **(A)** Weight difference compared to start weight during treatment (Mixed effect analysis followed by one-way ANOVA, Sidak’s multiple comparison test). **(B)** Tumor volume during treatment (8 mice/group) (Mixed effect analysis followed by one-way ANOVA, Sidak’s multiple comparison test). **(C)** Tumor weight at day 28 of treatment (13-15 tumors/group). **(D)** Immunohistochemistry analysis of Ki67 staining on tumor samples (8-12 tumors/group) and representative pictures of Ki67 staining (green) with DAPI counter staining (blue) per group: vehicle (V), inecalcitol (25 µg/kg) (I), palbociclib (50 mg/kg) (P) and combination (C). Scale bar = 50 µm. **(E)** P-Rb, total Rb (110 kDa) and p-CDK2 (33 kDa) protein expression in tumors from vehicle (V), inecalcitol (25 µg/kg) (I), palbociclib (50 mg/kg) (P) and combination (C) treated mice (6 tumors/group). One representative blot is shown. β-actin (42 kDa) was used as loading control. The ratio of phosphorylated to non-phosphorylated relative protein expression is indicated under the representative blot. Data are expressed as fold increase vs control (control value = 1). **(F)** Relative quantification of p-Rb, total Rb and p-CDK2 protein expression (5/6 tumors/group). Data shown as mean ± SD. **(G)** Serum (8 mice/group) and urine (3 pooled samples/group) calcium concentration. Data shown as mean ± SEM of one experiment (8 mice/group) ***p ≤ 0.001; ****p ≤ 0.0001 compared to vehicle; ^$$^p ≤ 0.01; ^$$$$^p ≤ 0.0001 compared to inecalcitol or palbociclib monotherapy (one-way ANOVA, Sidak’s multiple comparison test).

Next, we evaluated the combination treatment in a TNBC cell line-derived xenograft model of BT20 cells (**Figure 6A**). Because the tumor cells proliferate rapidly in this model, mice were treated for only 28 days to prevent tumors from exceeding the ethically approved tumor volume. The combination therapy did not induce weight loss during treatment (**Figure 7A**). After 28 days of treatment, the combination therapy was not able to reduce tumor volume significantly compared to vehicle, inecalcitol or palbociclib monotherapy (**Figure 7B**). Also, tumor weight was not changed after treatment with the combination therapy of inecalcitol and palbociclib (**Figure 7C**).

Evaluation of Ki67-positive cells in the tumor samples, demonstrated a significant 48% decrease in proliferating cells after treatment with palbociclib, while the combination therapy reduced the number of Ki67-positive cells by 24% compared to vehicle treatment (**Figure 7D**).

To evaluate the effect on cell cycle progression, total, p-Rb and p-CDK2 protein expression in the tumor samples were determined. Treatment with the combination therapy did not decrease the amount of total, p-Rb and p-CDK2 protein expression significantly. Also, the ratio of p-Rb/Rb protein levels was not altered by the treatment (**Figures 7E, F**). As previously shown in the ER^+^ xenograft model, we observed a significant increase in serum and urinary calcium concentrations in inecalcitol-treated mice and this increase was slightly more pronounced in the combination-treated group compared to inecalcitol-treated mice (**Figure 7G**). A possible explanation could be that palbociclib inhibits CYP24A1 activity, however, we did not find any differences in circulating 1,25(OH)_2_D_3_ concentrations in mice treated with palbociclib, arguing against this hypothesis (**Supplementary Figure 3B**).

Together, these *in vivo* data demonstrate a pronounced tumor inhibitory effect of the combination treatment with inecalcitol and palbociclib in ER^+^ MCF7 xenografts, whereas this treatment strategy did not prove to be effective in TNBC BT20 xenografts.

## 4. Discussion

Normal and malignant cell proliferation is governed by CDK4/6-mediated Rb phosphorylation, which subsequently allows E2F-mediated transcription of cell cycle genes, such as Cyclins A and E, which is critical for cell cycle progression. Interestingly, CDK4/6 inhibitors, such as palbociclib, have significant clinical utility for the treatment of ER^+^ HER2^-^ metastatic BC, especially when combined with blockers of ER-signaling (15–17). Since it was previously described that 1,25(OH)_2_D_3_ and its analogs possess potent anticancer properties in BC, the aim of this study was to investigate if the combination of the vitamin D_3_ analog inecalcitol with the clinically approved palbociclib enhances the effects of both single compounds in *in vitro* and *in vivo* BC models. As therapeutic options for TNBC are still very limited, the efficacy of this novel combination regimen was not only evaluated in ER^+^ BC cell lines (MCF7, T47D), but also in different TNBC cell lines (BT20, HCC1143, Hs578T).

The expression of a functional VDR is a prerequisite for the responsiveness of the BC cell lines to the antiproliferative effects of 1,25(OH)_2_D_3_. Indeed, mammary tumors derived from *Vdr*-null mice are resistant to the tumor-suppressive effects of 1,25(OH)_2_D_3_ (18). Furthermore, BC cell lines, which do not express the VDR, are not growth-retarded by 1,25(OH)_2_D_3_-mediated signaling (19). Therefore, we first confirmed that all BC cell lines examined expressed the VDR. The functionality of this VDR expression was demonstrated by elevated VDR protein levels and enhanced transcript levels of the catabolic enzyme *CYP24A1* and of known VDR target genes (*TRPV6*, *CAT*, *SLC37A2*, *IGFBP3*) after inecalcitol treatment.

In contrast to VDR expression, *CYP24A1* transcription appeared to be more strongly induced in the ER^+^ cell lines. Basal transcript levels of *CYP27B1*, responsible for the activation of 25(OH)D_3_, were not different between the different cell lines. While VDR signaling downregulates *CYP27B1* expression in the kidney (20), inecalcitol did not affect *CYP27B1* transcript levels in the BC cell lines, which is in agreement with earlier findings in 1,25(OH)_2_D_3_-treated MCF7 cells (21). Also, treatment with palbociclib did not affect transcript levels of the vitamin D metabolizing genes (data not shown).

Detailed dose-finding experiments in ER^+^ MCF7 and TNBC BT20 cells showed that palbociclib treatment was effective as monotherapy in both cell lines with EC_50_-concentrations around 10^-7^-10^-8^ M, whereas treatment with inecalcitol had significant antiproliferative effects in MCF7, but not in BT20 cells. Interestingly, combined treatment with inecalcitol and palbociclib potentiated the growth suppressive effect of palbociclib in both cell lines enabling the use of lower drug concentrations. Also in the other ER^+^ and TNBC cells lines examined, we observed that a combination of inecalcitol (10^-8^ M) and palbociclib (10^-7^ M) had superior antiproliferative effects than either compound alone, and this was not only observed in the [^3^H]thymidine incorporation assays but also in the IncuCyte analysis, which is based on the degree of cell culture confluencies. In both assays, the ER^+^ BC cell lines investigated in this study were more responsive to the growth-inhibitory effects of inecalcitol, which was not only evidenced by a stronger growth suppression but also by an earlier response to treatment. These data are in line with previous observations that showed an elevated response to vitamin D-signaling in ER^+^ versus ER^-^ BC cell lines (12). Other studies (15, 22, 23) suggested that ER^+^ BC cells are also more sensitive to the growth-suppressive effects of palbociclib. In our study, we did not observe an apparent increased susceptibility to palbociclib monotherapy in ER^+^ BC cell lines compared to the TNBC cell lines in the [^3^H]thymidine incorporation assay, but the IncuCyte data clearly demonstrated increased growth suppression of the ER^+^ BC cell lines compared to the TNBC cell lines by palbociclib.

Importantly, the combination of inecalcitol and palbociclib efficiently hampered tumor growth in a MCF7 cell line-derived xenograft model and was accompanied by a decreased number of proliferating cells. However, this growth reduction was associated with a significant alteration of calcium homeostasis, which appeared to be slightly more pronounced in the combination treatment. A possible explanation could be that palbociclib inhibits CYP24A1 activity, however, we did not find any differences in circulating 1,25(OH)_2_D_3_ concentrations in mice treated with palbociclib, arguing against this hypothesis. The *in vitro* finding that ER^+^ BC cells were more susceptible than TNBC cells to the growth inhibitory effects of the combination treatment was confirmed in an *in vivo* situation. Indeed, the combination of inecalcitol and palbociclib proved to be unsuccessful in slowing down tumor growth in a TNBC BT20 cell line-derived xenograft model.

Mechanistically, *in vitro* palbociclib monotherapy resulted in accumulation of cells in the G1 phase of the cell cycle in all BC cell lines examined, except in Hs578T TNBC cells, and this was accompanied by a decrease in S and/or G2/M phase cells, confirming earlier findings (22, 24–28). In contrast, inecalcitol monotherapy did not significantly affect cell cycle distribution in any of the cell lines, probably related to the rather low dose administered (10^-8^ M). Indeed, higher concentrations of 1,25(OH)_2_D_3_ and its analogs do regulate cell cycle progression by hampering the G1/S transition (2, 18, 29, 30). Yet, addition of this lower dose of inecalcitol to palbociclib treatment resulted in a more pronounced shift in cell cycle distribution than caused by palbociclib alone in all cell lines except in Hs578T cells. As p-Rb is a major gatekeeper of cell cycle progression, protein levels of (p)-Rb were investigated in MCF7 and BT20 cells. In ER^+^ MCF7 cells, palbociclib and inecalcitol monotherapy not only decreased Rb phosphorylation but also tended to decrease cellular Rb levels confirming previous observations (31–36). Again, inecalcitol potentiated the effect of palbociclib resulting in greatly reduced (p)-Rb levels. In the TNBC BT20 cell line, only p-Rb levels were significantly downregulated by the combination treatment, which may account for the smaller alterations in cell cycle distribution observed in this cell line. In line with these observations, phosphorylation of CDK2, a key player in G1/S transition, was decreased upon combined treatment with inecalcitol and palbociclib in MCF7 and BT20 cells, whereas total CDK2 levels were only decreased in MCF7 cells. The same mechanism of inhibiting cell cycle progression, by downregulation of (p)-Rb and (p)-CDK2, contributed to the *in vivo* antitumor effect of the combined inecalcitol and palbociclib therapy in MCF7-derived xenografts.

As shown in previous publications, palbociclib monotherapy induced protein levels of Cyclin E1 in MCF7 and BT20 cells, probably as part of a survival mechanism activated to bypass cyclin D1-CDK4/6 dependence (27, 37). Remarkably, addition of inecalcitol to palbociclib treatment tended to decrease the palbociclib-mediated induction of Cyclin E1 in MCF7 cells, whereas it had no effect or tended to further increase Cyclin E1 expression in BT20 cells, which may favor cell proliferation in the latter cells (**Supplementary Figure 4**) (38). As the CDK-inhibitor p21 is reported as a primary 1,25(OH)_2_D_3_-target gene, p21 protein levels were quantified (data not shown). Unlike other studies we did not observe induction of p21 by inecalcitol. Differences in concentrations applied (10^-7^ M versus 10^-8^M in our study) and/or timing (24 h versus 72 h in our study) may be responsible for these discordant findings. Also, p21 levels were not affected by palbociclib monotherapy or after combination therapy, while other studies in which higher palbociclib concentrations were applied, detected upregulation of p21 (35, 36). However, a recent study demonstrated that the CDK-inhibitors p21 and p27 are not required for palbociclib-mediated cell cycle arrest (39).

Interestingly, the combination of inecalcitol and palbociclib in MCF7 and BT20 cells significantly induced oxidative stress, an additional stress response associated with cell cycle arrest and apoptosis. Also, the combination treatment upregulated mitochondrial content in both cell lines. Yet, previous studies illustrated that a higher concentration of palbociclib (10^-6^ M, 6 days) increased cellular ROS levels in MCF7 and T47D cells, leading to the development of cellular senescence (33). However, in our study, we were unable to detect cellular senescence in any of the experimental conditions (data not shown). Yet treatment with inecalcitol, alone or in combination with palbociclib, resulted in apoptotic cell death, which was associated with PARP cleavage, confirming previous studies showing that inecalcitol induced apoptotic cell death in MCF7 cells (31) and PARP cleavage in squamous cell carcinoma (40). Palbociclib monotherapy, however, did not cause apoptotic cell death in MCF7 or BT20, which is in line with the results of Vijayaraghavan et al. in ER^+^ T47D and MCF7 cells (33). In contrast, in hepatocellular carcinoma, palbociclib induced apoptosis not by cleavage of PARP but by activation of 5′ AMP-activated protein kinase and inhibition of protein phosphatase 5 (41).

The PI3K/Akt/mTOR signaling pathway has a major role in essential cellular activities, such as cell proliferation and apoptosis, and is often deregulated in breast cancer (42, 43). Interestingly, treatment with 1,25(OH)_2_D_3_ or its analogs is able to suppress this pathway in multiple cell types, including BC cells, resulting in decreased phosphorylation of the PI3K/Akt/mTOR-downstream targets P70S6K and 4E-BP1, thereby inhibiting ribosomal translation and protein synthesis (44–47). Also, palbociclib therapy is able to decrease mTOR activation and inhibits P70S6K phosphorylation (48–50). In contrast, other studies reported that palbociclib activates the mTOR pathway (27, 36). The reason for these discrepant findings could be due to differences in compound concentrations, timing and cell lines (ER^+^ vs TNBC) investigated. Whereas (p)-Akt was not affected by inecalcitol or palbociclib, opposite effects on (p)-mTOR signaling were observed in MCF7 and BT20 cells. Indeed, the combination treatment resulted in decreased (p)-mTOR protein levels in BT20 cells, which was accompanied by decreased phosphorylation of P70S6K and 4E-BP1. Surprisingly, however, in MCF7 cells, (p)-mTOR as well as (p)-4E-BP1 protein levels were elevated after inecalcitol or palbociclib monotherapy and after the combination therapy, indicating that in these cells the combination treatment is highly efficient in blocking cell cycle progression in the presence of activated mTOR signaling. These results are in line with recent observations in MCF7 cells, where CDK4/6 inhibition blocked cell cycle progression and reduced Rb phosphorylation in the presence of deregulated mTOR signaling, which resulted in elevated mitochondrial content and increased ROS production (36, 51).

In summary, our findings suggest that addition of a potent vitamin D_3_ analog such as inecalcitol to palbociclib treatment enhances the antitumor effects of palbociclib monotherapy in ER^+^ MCF7 cells and xenografts. In this model, inecalcitol and palbociclib combination therapy reduced cell proliferation by inhibition of Rb while mTOR activity was sustained. It would be interesting to further explore the effect of mTOR inhibition using a pharmacological compound everolimus in combination with inecalcitol and palbociclib treatment in MCF7 cells. As everolimus is used in the clinic for metastatic *PIK3CA*-mutated ER^+^ BC, the triple combination might enhance the antiproliferative effect of the single compounds. In addition, it would be useful to test the inecalcitol and palbociclib combination therapy with hormonal therapy such as tamoxifen or fulvestrant to further explore the clinical relevance of this treatment strategy.

In contrast, inecalcitol and palbociclib combination therapy was less effective in slowing down *in vitro* cell growth in different TNBC models and was not able to retard tumor growth in a BT20-derived TNBC xenograft model.

Although the exact mechanism for the difference in responsiveness between ER^+^ and TNBC cells needs to be further elucidated, this study provided novel insights in the pathways affected by inecalcitol and/or palbociclib treatment in different BC subtypes.

## Data Availability Statement

The original contributions presented in the study are included in the article/**Supplementary Material**. Further inquiries can be directed to the corresponding author.

## Ethics Statement

The animal study was reviewed and approved by Ethical Committee for Animal Experimentation KU Leuven (P200/2017).

## Author Contributions

JV, LV, SL, SS, SD, HW and AV conceptualized and designed the study. JV, SB and IJ performed experiments and acquired data. JV and LV performed analysis and interpretation of the data. JV, LV, HW and AV wrote the manuscript. All authors contributed to the article and approved the submitted version.

## Funding

JV received a PhD fellowship Fundamental Research from Flanders Research Foundation (FWO; 11C2921N). SL received a PhD fellowship Strategic Basic Research from Flanders Research Foundation (FWO; 1S46318N). SS was a senior postdoctoral research fellow of the Flanders Research Foundation (FWO; 12H5917N). HW was senior clinical investigator of the Flanders Research Foundation (FWO-Vlaanderen). AV received funding from the University of Leuven (C16/18/006) and Flanders Research Foundation (FWO; G.0D01.20N, G0D4217N).

## Conflict of Interest

The authors declare that the research was conducted in the absence of any commercial or financial relationships that could be construed as a potential conflict of interest.

## Publisher’s Note

All claims expressed in this article are solely those of the authors and do not necessarily represent those of their affiliated organizations, or those of the publisher, the editors and the reviewers. Any product that may be evaluated in this article, or claim that may be made by its manufacturer, is not guaranteed or endorsed by the publisher.
