# Supplementary material for: The Combination of the CDK4/6 Inhibitor, Palbociclib, With the Vitamin D3 Analog, Inecalcitol, Has Potent In Vitro and In Vivo Anticancer Effects in Hormone-Sensitive Breast Cancer, But Has a More Limited Effect in Triple-Negative Breast Cancer
Source: Front Endocrinol (Lausanne). 2022 Jun 17;13:886238. doi: 10.3389/fendo.2022.886238 (PMC9248359; doi:10.3389/fendo.2022.886238)
Supplement: Supplementary file 1 [file DataSheet_1.docx]

## **Supplementary Materials and Methods**

**1 Serum and urine biochemistry**

Serum and urinary calcium (OSR60117) concentrations were measured on an AU640 chemistry analyser (Beckman Coulter). Levels of 25(OH)D_3_ and 1,25(OH)_2_D_3_ were measured by LC-MS/MS at the University Hospital of Leuven

**2 Micro-computed tomography (µCT)**

Ex vivo μCT images were taken on the high resolution Skyscan 1172 system (Bruker, Kontich, Belgium). The scanner source was set at 50 kV and 200 μA and a 0.5 mm aluminium filter was applied. Scans were taken with a pixel size of 5 μm with an angular increment of 0.4 degrees and a frame averaging of 2. Serial tomographs were reconstructed from raw data with the filtered Feldkamp cone-beam method (NRecon software, Bruker) and analysis was performed using the CT Analyzer software (CTAn, Bruker) with global thresholding. The global threshold was visually determined to optimally separate the bimodal histogram into bone and soft tissue. 3D morphometric analysis was performed on the tibia in manually selected trabecular and cortical volumes of interest (between 1 to 2.5 mm distal from the growth plate to assess trabecular parameters and between 2.5 and 3 mm to calculate cortical parameters). Analysis was performed according to the guidelines of the American Society for Bone and Mineral Research (1). 3D models were constructed with CTvox software (Bruker).

**3 References**

1. Bouxsein ML, Boyd SK, Christiansen BA, Guldberg RE, Jepsen KJ, Muller R. Guidelines for Assessment of Bone Microstructure in Rodents Using Micro-Computed Tomography. *J Bone Miner Res* (2010) 25(7):1468-86. Epub 2010/06/10. doi: 10.1002/jbmr.141.
