# Supplementary material for: The Combination of the CDK4/6 Inhibitor, Palbociclib, With the Vitamin D3 Analog, Inecalcitol, Has Potent In Vitro and In Vivo Anticancer Effects in Hormone-Sensitive Breast Cancer, But Has a More Limited Effect in Triple-Negative Breast Cancer
Source: Front Endocrinol (Lausanne). 2022 Jun 17;13:886238. doi: 10.3389/fendo.2022.886238 (PMC9248359; doi:10.3389/fendo.2022.886238)
Supplement: Supplementary file 2 [file DataSheet_2.docx]

## **Supplementary figures**

**Supplementary Figure 1**

**
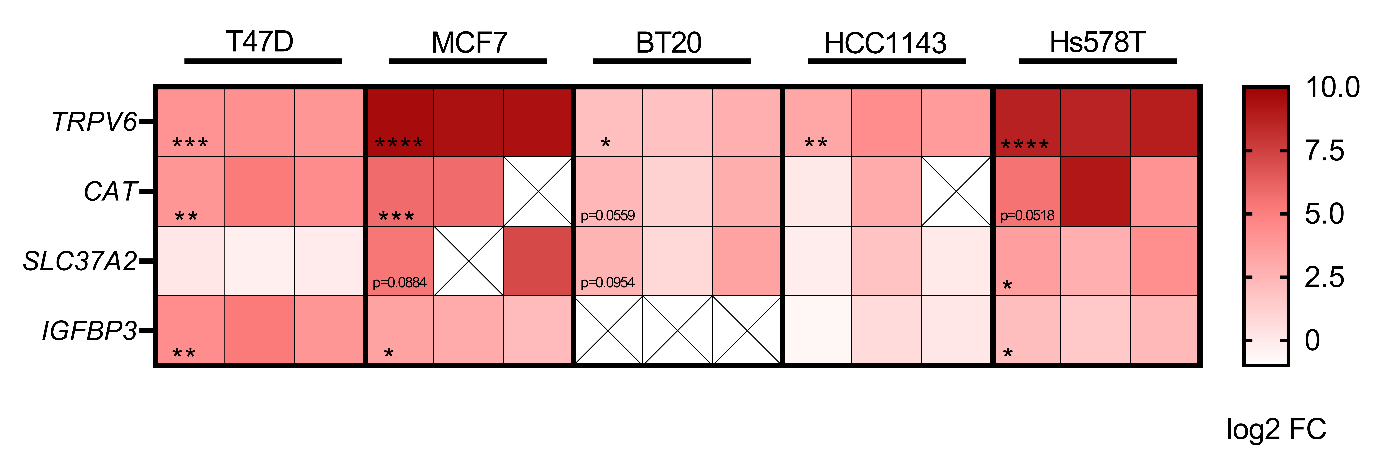
Supplementary Figure 1: Regulation of known VDR target genes by inecalcitol in BC cell lines.** *TRPV6*, *CAT*, *SLC37A2* and *IGFBP3* mRNA expression was determined in T47D, MCF7, BT20, HCC1143 and Hs578T cells after treatment with vehicle (EtOH) or inecalcitol (10^-8^M, 72 h). Expression levels were normalized for *GAPDH* expression and fold change (FC) values (inecalcitol- versus vehicle-treated cells) were log2 transformed and depicted (n=3, performed in duplicate, each replicate is represented by a square). Crossed squares indicate samples where FC could not be determined due to undetectable gene levels in vehicle-treated samples. Significant changes in log2 FC of the three biological replicates are indicated in the first square; *p≤0.05; **p≤0.01; ***p≤0.001; ****p≤0.0001 (one sample t-test).

**Supplementary Figure 2**


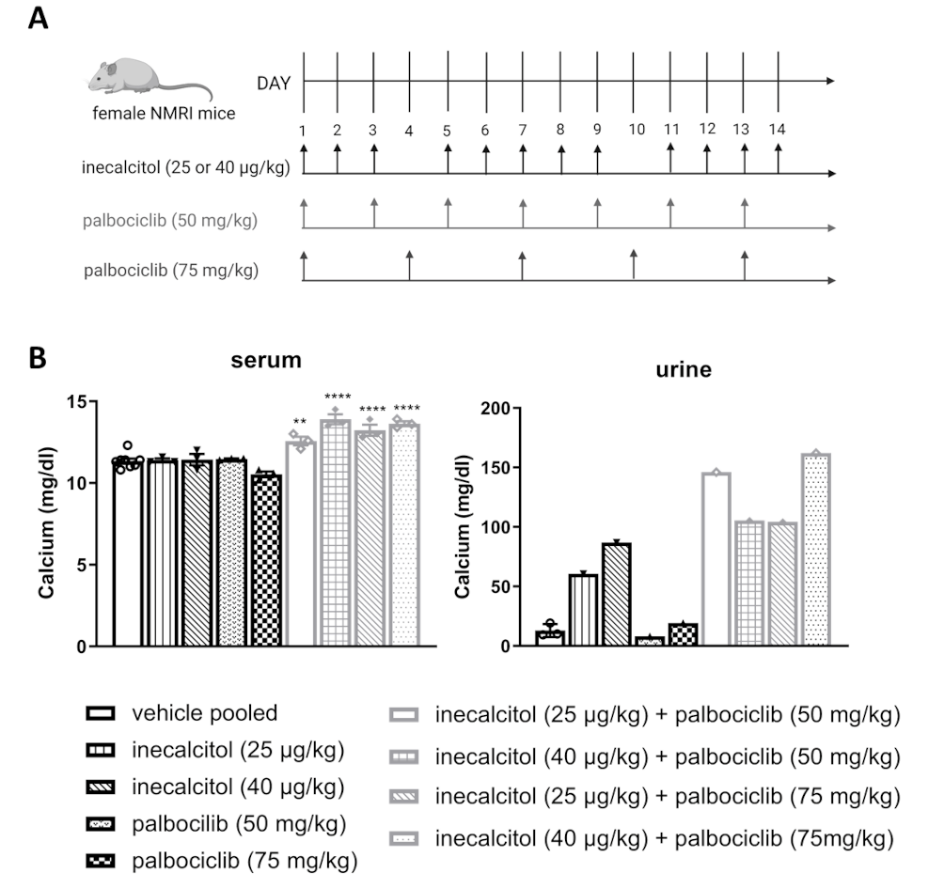


**Supplementary Figure 2: Experimental dose titration study to determine the optimal dose for inecalcitol and/or palbociclib treatment. (A)** Overview of experimental setup of the dose titration study. Created with BioRender.com. **(B)** Calcium analysis of serum (3 mice/group) and urine (1 pooled sample/group). Data shown as mean ± SEM of one experiment (3 mice/group). **p≤0.01; ****p≤0.0001 compared to vehicle (one-way ANOVA, Sidak’s multiple comparison test).

**Supplementary Figure 3**


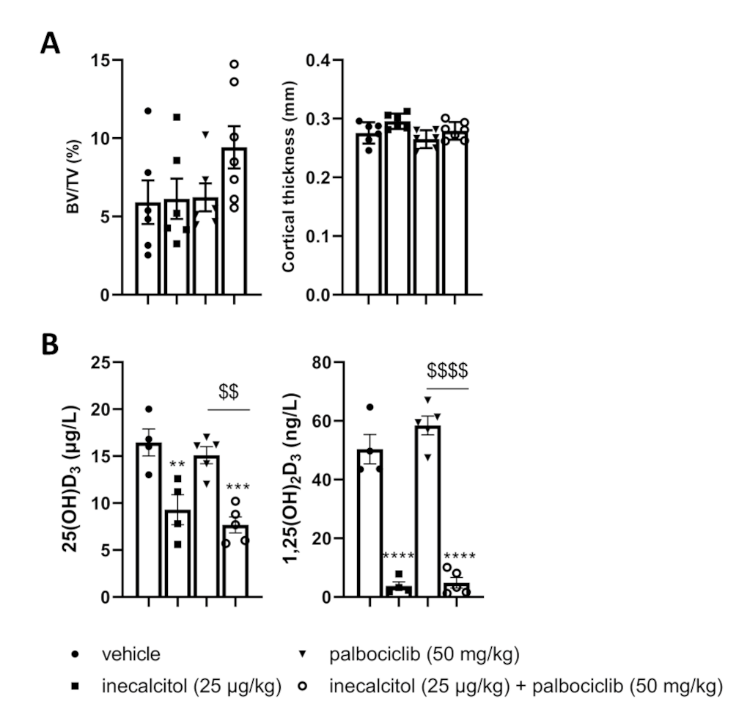


**Supplementary Figure 3: bone and serum analysis of MCF7 and BT20 xenografts after treatment with inecalcitol and/or palbociclib. (A)** MicroCT analysis of MCF7 xenografts after 49 days of treatment. No significant difference between bone volume and cortical thickness was observed (6-7 mice/group). **(B)** Serum analysis of 25(OH)D_3_ and 1,25(OH)_2_D_3_ in BT20 xenografts after 28 days of treatment (4-5 mice/group). Data shown as mean ± SEM of one experiment (8 mice/group). **p≤0.01; ***p≤0.001; ****p≤0.0001 compared to vehicle; $$p≤0.01; $$$$p≤0.0001 compared to inecalcitol or palbociclib monotherapy (one-way ANOVA, Sidak’s multiple comparison test).

**Supplementary Figure 4**

**
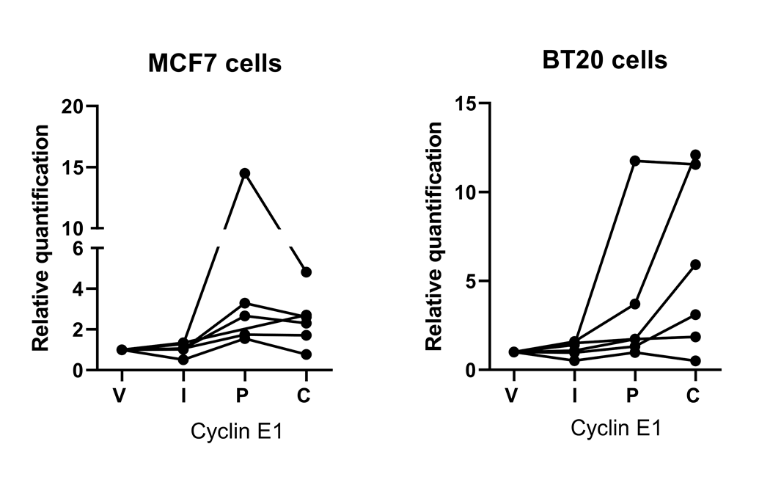
**

**Supplementary Figure 4: Effect of inecalcitol and/or palbociclib treatment on Cyclin E1 expression in MCF7 and BT20 cells.** Relative expression levels of Cyclin E1 in MCF7 and BT20 cells after treatment with V = vehicle (EtOH), I = inecacitol (10^-8^ M), P = palbociclib (10^-7^ M) and C = combination of inecalcitol (10^-8^ M) and palbociclib (10^-7^ M) (n= 5-6).
